# The Relationship Between Cognitive Resource Consumption During Gameplay and Postgame Aggressive Behaviors: Between-Subjects Experiment

**DOI:** 10.2196/48317

**Published:** 2023-11-14

**Authors:** Huina Teng, Lixin Zhu, Xuanyu Zhang, Boyu Qiu

**Affiliations:** 1 School of Health Management, Guangzhou Medical University, Guangzhou, China; 2 School of Mental Health, Guangzhou Medical University, Guangzhou, China

**Keywords:** video games, hurting, helping, cognitive resources, aggressive behaviors

## Abstract

**Background:**

The question of how video games can shape aggressive behaviors has been a focus for many researchers. Previous research has focused on how violent video game content leads to postgame aggressive behaviors. However, video games not only convey violence or prosocial content to players but also require cognitive effort from individuals. Since human cognitive resources are limited, consuming more cognitive resources in a game leads to less cognitive resources to suppress aggressive impulses. Therefore, the depletion of cognitive resources from playing video games may also lead to changes in postgame aggressive behaviors.

**Objective:**

This study aimed to examine the relationship between cognitive resources consumed in video games and postgame aggressive behaviors.

**Methods:**

A total of 60 participants (age: mean 20.22; range 18-24 y) were randomly assigned to either the high-load group or the low-load group. Participants from both groups played a video game centered around college life. In the low-load group, participants followed the gameplay instructions to complete it. In the high-load group, participants were given an extra digital memory task to complete while playing the game. Participants in both groups played the video game for about 25 minutes. A maze selection task was then conducted to measure the participants’ helping and hurting behaviors.

**Results:**

The independent samples 2-tailed *t* tests showed that the high-load group had significantly higher hurting scores (mean 3.13, SD 2.47) than the low-load group (mean 1.90, SD 2.12; *t*_58_=−2.07, *P*=.04; Cohen *d*=−0.535), whereas helping behaviors were not significantly affected (*t*_58_=1.52, *P*=.13; Cohen *d*=0.393).

**Conclusions:**

As more cognitive resources are consumed in a video game, more hurting behaviors are exhibited after the game. This finding proposes an alternative route by which video games impact aggressive behaviors, adding to previous theories and raising concerns about the popularity of cognitive training games.

## Introduction

Video games have become popular as a form of entertainment [1]. The General Aggression Model [2] and the General Learning Model [3] suggest that violent content in video games influences aggressive cognition [4,5] and negative emotions [6,7], leading to aggressive behaviors [8,9]. However, individuals passively receive content delivered by the game and actively invest cognitive resources while playing video games, potentially affecting social behaviors in the short term.

Two possible explanations were initially used to study honest behaviors [10,11], which help us understand the relationship between cognitive resource consumption and social behavior. The “Will” hypothesis [12] suggests that individuals have a natural tendency to engage in negative behaviors (eg, cheating) and therefore need to expend cognitive resources to overcome the impulse to engage in negative behaviors. The “Grace” hypothesis [13] assumes that individuals are naturally inclined to engage in positive behaviors (eg, honesty) and that individuals need to expend cognitive resources to overcome the original impulse so that they can behave negatively to gain benefits (eg, cheating).

Both aggressive (eg, hurting) and prosocial (eg, helping) behaviors are regulated by human cognitive processes [2,3] and are closely linked to cognitive load. According to social learning theory [14], both aggressive and prosocial behaviors are acquired by observing and imitating others. This position suggests that cognitive elements such as attention [15], executive function [16], and working memory [17] impact aggressive and prosocial behaviors and are hindered by cognitive load. Therefore, these 2 hypotheses (the “Will” and “Grace” hypotheses) concerning honest behavior based on decreased cognitive resources may be equally beneficial in elucidating both hurting and helping behaviors.

Based on the aforementioned reasoning, the “Will” hypothesis suggests that when individuals use excessive cognitive resources while gaming, they may lack sufficient cognitive resources to control the impulse and therefore may display more hurting behaviors. Conversely, the “Grace” hypothesis posits that if cognitive effort is extended in the video game, the individual may lack sufficient cognitive resources to restrain the initial impulse and subsequently display more helping and less hurting behaviors. At present, it remains unclear as to which outcome transpires from expending cognitive resources in gaming.

This study examines the relationship between cognitive resource consumption in video games and postgame aggressive behaviors. If the “Will” hypothesis is met, consuming more cognitive resources during video gaming will lead individuals to show more aggressive behaviors after the game. If the “Grace” hypothesis is met, consuming more cognitive resources during gaming will lead individuals to show less postgame aggressive behaviors.

## Methods

### Participants

Participants were recruited through a college social media platform (ie, WeChat groups for college students). The participants were screened based on the following criteria: (1) no history of mental disorders, (2) normal or corrected-to-normal vision, and (3) no prior experience with the task used in this study. A total of 63 participants were recruited, and 3 of them did not show up, resulting in a final sample of 60 college students.

An a priori power analysis using G*Power (version 3.1.9.7; Heinrich-Heine-Universität Düsseldorf) was conducted to estimate the sample size. Based on data from Yin et al [18] (n=40), which compared 2 independent groups on the same task, the minimum effect size in that study was 0.77. With a significance level of α=.05, Cohen *d*=0.77, and power=.80, the minimum sample size needed with this effect size was 56 for the independent samples 2-tailed *t* tests used in this study. Therefore, the obtained sample size of 60 was more than adequate to test the hypothesis.

The 60 participants were randomly assigned to the high-load group (n=30, 50%; 11/30, 37% female; age: mean 20.47, range 18-24 y) and the low-load group (n=30, 50%; 12/30, 40% female; age: mean 19.97, range 18-24 y). Informed consent was obtained from each participant before the experiment.

### Measures

#### Video Games

The study used a video game adapted from the one used by Yin et al [18], where participants played the role of a first-year college student facing diverse situations based on college life. The content that the participants encountered in the scenarios was neutral (ie, not including hurting or helping behavior, to avoid in-game hurting or helping content from affecting postgame maze choices) and related to daily life at college, such as choosing which clubs to join, what they chose to wear out, and what sports they were involved in after class. In the low-load group, participants followed the game instructions to complete it. In the high-load group, participants had an additional digital memory task while playing the game. They had to memorize a 4-digit “password” and enter it after completing 5 situations. Subsequently, they received a new password and had to enter it after 5 further situations. In 60 situations, they were given the “password” 12 times, and throughout the game, they had to keep a 4-digit number in their memory. In the high-load group, participants could not take notes while trying to remember the password. Thus, they had to memorize it internally and update the numbers after every 5 situations. They were told to prioritize remembering the password correctly and follow the game instructions to finish it, thus requiring more cognitive effort and resources than the low-load group. On average, the participants completed the game in approximately 25 minutes.

#### Maze Selection Task

The maze selection task used in the study was modified from the Tangram Task, which is commonly used in psychological research on video games [8,19,20]. Notably, the results of the Tangram Task have been found to correlate significantly with aggression and prosocial behaviors measured through scales and laboratory tasks [21]. In some countries, students may be unfamiliar with tangrams, so Yin et al [18] suggested using mazes instead to measure helping and hurting behaviors.

In the maze selection task, participants were required to choose 11 mazes for the next participant (who is fictional) from 30 given mazes. The fictional participant would walk the maze using paper and pencil, and if they could walk at least 10 mazes in 10 minutes, the fictional participant would be paid an additional ¥30 (US $4.12). There were 10 mazes based on 5 × 5 cells (small and easy), 10 based on 10 × 10 cells (medium), and 10 based on 20 × 20 cells (large and challenging). Participants could help the following participant by selecting easier mazes or hurt them by selecting difficult mazes. The helping score was the total number of easy mazes chosen, and the hurting score was the total number of difficult mazes chosen. Notably, higher scores indicated more helping or hurting behaviors.

### Analyses

Only 1 of the 30 participants in the high-load group misremembered the “password” in 1 response, whereas a total of 360 responses (30 participants in the high-load group × 12 responses per participant) were recorded during the experiment. The incorrect response accounted for 0.3% (1/360) of the total responses in the high-load group. Therefore, it was concluded that having only 1 incorrect response was acceptable and that the participants in the high-load group retained their memory for the 4-digit number throughout the experiment. The analysis of the data then included all 30 participants in the high-load group, including the 1 who incorrectly remembered once.

After completing the data collection, we conducted independent samples 2-tailed *t* tests using the helping and hurting scores from the maze selection task as dependent variables and groups (high-load and low-load groups) as independent variables. In the pretests for equality of variances, the samples passed the Levene test for homogeneity of variances (helping scores: *F*_1,58_=0.03, *P*=.87; hurting scores: *F*_1,58_=0.52, *P*=.48). Therefore, the Student *t* test was used to calculate the statistical probability (*P* value), and Cohen *d* was used to indicate the effect size.

### Ethical Considerations

The study was approved by the Research Ethics Committee of South China Normal University (#SCNU-2021-275). Written informed consent was obtained from each participant. Our data underwent deidentification processes. Participants received ¥30 (US $4.12) as compensation.

## Results

Descriptive statistics for the helping and hurting scores from the maze selection task are shown in Table 1. The independent samples 2-tailed *t* tests showed a significant difference between the 2 groups in hurting scores (*t*_58_=−2.07, *P*=.04; Cohen *d*=−0.535) but not helping scores (*t*_58_=1.52, *P*=.13; Cohen *d*=0.393). The high-load group had significantly higher hurting scores (mean 3.13, SD 2.47) than the low-load group (mean 1.90, SD 2.12; *P*=.04).

**Table 1.** Descriptive statistics for the helping and hurting scores from the maze selection task. The helping score was the total number of easy mazes (based on a 5 × 5 cell) participants chose in the maze selection task, and the hurting score was the total number of difficult mazes (based on a 20 × 20 cell) they chose, with higher scores indicating more helping or hurting behaviors.

Score	High-load group, mean (SD)	Low-load group, mean (SD)
Helping	2.07 (2.41)	2.97 (2.17)
Hurting	3.13 (2.47)	1.90 (2.12)

## Discussion

### Principal Findings

The main finding of this study is that investing more cognitive resources in video gaming led to significantly more postgame hurting behaviors (*P*=.04), whereas helping behaviors were not significantly affected (*P*=.13). Consistent with the “Will” hypothesis [12], individuals in this study may be more inclined to exhibit aggressive behaviors, and inhibiting aggressive behaviors requires a drain on cognitive resources. When cognitive resources are consumed during play, individuals may not have sufficient cognitive resources to inhibit aggressive behaviors and therefore tend to exhibit more aggressive behaviors after gaming.

### Comparison to Prior Work

Over the past 2 decades, research on the influence of video games on aggressive behavior has relied heavily on the General Aggression Model [2], suggesting that the violent content in video games is a key factor in causing individuals to exhibit more aggressive behavior. The results of this study complement this model by suggesting another possible cause of more aggressive behavior, namely, the consumption of excessive cognitive resources in games. In actual play, these 2 processes may alter aggressive behaviors simultaneously. The violent content of the game shapes individuals’ aggressive scripts and schemas [2]. In contrast, a large amount of cognitive input in the game leaves individuals without sufficient cognitive resources to suppress their impulses to hurt.

This result also raises concerns about cognitive training in video games. In recent years, the use of video games for cognitive training has become increasingly popular [22]. Video games are easy to access and fun, and participants are more willing to participate regularly in video game–based cognitive training [23,24]. Numerous studies have shown that video game training improves individuals’ visual attention [25,26] and executive functions [27-29]. However, the results of this study suggest that excessive cognitive resources spent on games may harm individuals’ social behaviors. Therefore, further research is urgently needed to assess how cognitive training games affect individuals’ aggressive behaviors.

It is important to state that this study provides evidence solely for the potential effect of cognitive load in gameplay on postgame hurting behaviors, and one must not infer directly from this outcome that cognitively training games inevitably result in long-lasting aggression. In most laboratory studies of video games [4,15,18-20], measures of social behavior are typically completed within 20-30 minutes after the game. Changes in social behavior observed under such conditions are generally transient and can be recovered after a period of time. This is also true in high-load games, where participants may feel cognitively exhausted for a short period of time after the game and are more likely to display aggression, but not irreversibly.

However, models of aggression and learning have been proposed specifically for this scenario. Anderson et al [2] note that while the temporary nature of the postgame effect is acknowledged, prolonged and repeated exposure to this state through extended gameplay can permanently alter an individual’s levels of aggression or prosocial tendencies. Therefore, although the laboratory findings indicate a short-term aftereffect of playing video games, it still warrants careful consideration and attention.

In addition to the duration of aftereffects and long-term effects, several factors were not tested in a single experiment. For example, it is unclear whether all highly cognitively demanding games induce aggression or only certain types of games. It is also uncertain whether the effect of cognitive load differs from the effect of violent game content and whether these effects interact. Further research is needed to investigate these aspects.

### Limitations

This study has certain shortcomings. First, it has been suggested that some cognitive factors such as attentional bias to different information also influence social behaviors [15], and it is still unknown whether these factors interact with the cognitive resources consumed in video games. The interaction of these factors with the consumption of cognitive resources during gameplay could be further tested, which would help to deepen our understanding of the mechanisms by which cognitive resources influence postgame social behaviors.

Second, only 1 task, the maze selection task, was used to measure helping and hurting behaviors, and multiple tasks measuring aggression and prosocial behaviors are needed to test this effect. The maze selection task is founded on a localized adjustment of the Tangram Task, which has undergone extensive use in numerous studies. Furthermore, the modified task has obtained validation through research that establishes its efficacy in measuring helping and hurting behaviors. Therefore, we believed that the results in this study are reliable. However, if multiple tasks measuring aggression and prosocial behaviors were used, it is possible to compare whether different types of social behaviors can all be affected by the in-game consumption of cognitive resources.

Third, the manipulation of cognitive resources in the game was achieved by increasing the working memory load. Therefore, it remains to be tested whether the same results can be obtained by depleting cognitive resources in other ways (eg, inhibition or switching ability). Investigating different manipulation of cognitive resources in future studies will aid in determining whether different types of cognitive depletion affect postgame social behaviors equally.

### Conclusion

This study highlights the negative impact of excessive cognitive resources spent on video games, potentially related to increased postgame aggressive behavior due to insufficient cognitive resources for inhibiting aggressive impulses. This finding proposes an alternative route by which video games impact aggressive behaviors, adding to previous theories and raising concerns about the popularity of cognitive training games.
